# Why We Conform

**DOI:** 10.1371/journal.pbio.1000277

**Published:** 2010-02-02

**Authors:** Julia Fischer

**Affiliations:** Cognitive Ethology Laboratory, German Primate Center, Göttingen, Germany

## Abstract

Are humans fundamentally helpful, or does coercion inevitably come with altruism? Julia Fischer examines this question in her review of Michael Tomasello's new book, *Why We Cooperate*.

**Figure:**
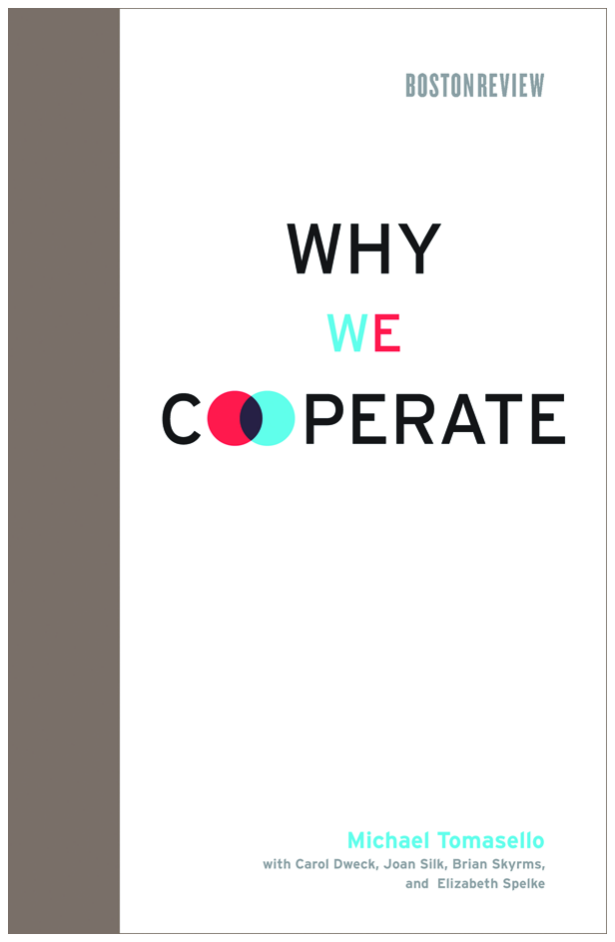
Tomasello, M with Carol Dweck, Joan Silk, Brian Skyrms, Elizabeth Spelke (2009) Why we cooperate. Boston, MA: MIT Press. 208 p. ISBN (cloth): 978-0-262-01359-8 US$14.95.

What makes us human, what sets us apart from other animal species, and which traits do we share with our closest living relatives? Ever since Darwin introduced the notion of continuity in his theory of evolution, humans have been obsessed with the question of how to distinguish themselves from all other species. In the postwar period, our species became known as “Man the Toolmaker,” until in the 1960s Jane Goodall watched chimpanzees using sticks to fish for termites, and that was that. We then distinguished ourselves using the term “Man the Hunter,” but the discovery that chimpanzees and other social carnivores engage in coordinated hunts refuted this type of collective action as the one decisive feature. More recently, the issue of culture has entered center stage. Trying to distinguish the cultural “haves” from the “have-nots” tends to generate more heat than light, and it seems much more productive to think about the cognitive prerequisites for social learning, attribution of mental states, and symbolic communication.

In his book *Why We Cooperate*, Michael Tomasello explores the socio-cognitive mindset that forms the basis of human sociality, including the creation of cultural artifacts and social institutions. The key message is that humans are fundamentally helpful and cooperative, as evidenced by infants' willingness to provide information, help, and share worldly goods. Later in life, experience may corrupt this benevolent attitude, but the core point for Tomasello is that children exhibit other-regarding preferences, and it is precisely this feature that sets them apart from our closest living relatives, the great apes.

Interest in the evolution of cooperation and altruism and the proposition that individuals do care about the well-being of others are testimony to the major paradigm shift in the current conception of the evolution of social behavior. At the height of radical Neo-Darwinism, individuals were seen as manipulators who benefited from modifying the behaviors of others. Suspicion would be a particularly useful attitude in an environment full of bluffing, cheating, and free-riding. Over the years, it has become clear that this view did not cover many of the complexities of human or other animal social life. Recently, however, we have seen a resurgence of the concept of “group selection,” this time not in the simplistic version that animals or humans act for the good of the group, but that they do so because it is in their own interest. If you live in a successful group, this will increase your own chances of survival. Thus, the argument goes, selection takes place at multiple levels, namely at the level of the gene, the individual, and the group [1]. The issue, however, is far from settled, and an alternative view is that kin selection suffices to explain the emergence of cooperation in groups [2].

The concise nature of *Why We Cooperate* makes it a perfect companion for a train ride. It is based on the Tanner Lectures that Tomasello gave last year at Stanford University. Tomasello is co-director of the Max Planck Institute for Evolutionary Anthropology in Leipzig, Germany and head of the Department of Developmental and Comparative Psychology. Over the last two decades, he and his collaborators have rolled out an impressive research program that centers on experimental testing of children and captive great apes. As Tomasello freely admits, the book is heavily biased towards his own work. There are two main chapters; in the first, he turns to the building blocks of cooperative behavior—sharing, helping, and providing information. He examines these from a comparative perspective and provides ample evidence that children go out of their way to come to the aid of a stumbling experimenter or point out to her the location of a missing tool, while chimpanzees rarely appear to grasp the situation. The second chapter extends the argument to the fundamentals of collaborative action (the so-called “we-intentionality” [3]), explores possible evolutionary scenarios for the emergence of this specific human attitude and examines the formation of social norms and institutions. The arguments flow nicely, and Tomasello knows how to capture his audience. Though at first there is a feeling that it all adds up too well, what really makes this book such a stimulating piece is the commentary section, in which four eminent scientists from neighboring fields probe alternative perspectives.

Joan Silk stresses the importance of the multidisciplinary approach. Game theory, for instance, has proven a useful tool in the analysis of evolutionary scenarios. Her main point is that, in collaborative endeavors, interests are typically not fully aligned (but not completely divergent, either). Silk is an anthropologist who has studied primates not only in the wild and in the lab, but also in meeting rooms. As she notes, anyone who has ever served on a committee has experienced firsthand how to grapple with misaligned interests.

Carol Dweck, a developmental psychologist, critically examines the notion that one-year-old children constitute “naked savages.” She makes the important point that child socialization begins at birth and that children very early on begin to form hypotheses about what is expected of them (see [4] for a beautiful account of cross-cultural differences in babies' first years). I found this perspective particularly important, as the majority of Tomasello's work is with white middle-class children whose parents think that it is a good idea to get them tested. Of interest may be how sensitive these children are to the situation. For instance, is their behavior influenced by an understanding of the experimenters' expectations? While it is commendable that cross-cultural studies are under way, it would be beneficial if these experimental tests were complemented by observational studies in order to explore what infants in different cultures actually do in their real lives.

The philosopher of science Brian Skyrms provides a number of examples where cooperation has evolved in species without a mind, such as bacteria. Of course, psychologists may dismiss the study “cooperative bio-film production in bacteria” precisely because these creatures have no brains. These cases may, however, help to identify the minimal requirements needed for cooperative behavior.

Elizabeth Spelke, also an eminent developmental psychologist, suggests that there is some “core knowledge” about the physical and social properties of the world that is shared across cultures and, to some extent, across species. She argues that language is the means by which children learn to relate different representational formats and combine them productively. So the question is whether language gives rise to shared intentionality and other forms of elaborate attribution of mental states, as she would argue, or if Tomasello is right in arguing that joint attention and shared intentionality (in some crude form) come first and pave the way for language. The answer is still up for grabs.

Despite its modest format, the book provides ample food for thought and could well be used as a starting point in discussion rounds and seminars. I would issue a warning however, to be aware of the limitations when comparing adult captive apes with young of our own species [5]. There is also an implicit connotation that chimpanzees constitute models for the last common ancestor of chimps and humans, which should be taken with a pinch of salt. This is not to say these comparative analyses are of no value; after all, chimpanzees and bonobos are our closest living relatives.

So are we much better than we often think we are? Are we really “born (and bred) to help,” as the title of the first chapter suggests? Tomasello points out that a correlate of the “we-intentionality” is to value conformity. From a very early age children do not only try to comply, they also make an effort to get others to comply as well. Humans have evolved emotional responses to violations of social norms, such as guilt and shame; we actively teach and have invented a frightening array of methods to punish and torture. Tomasello advances the view that the specific skills and motivations, which make us help and share, evolved in times of cooperative hunting. While I am not a great friend of drafting evolutionary tales of how or why certain traits evolved, I would argue that these skills are equally likely to have evolved in a setting of fierce between-group competition. We will probably never know. The overriding conclusion, however, is that cooperation and competition mutually depend on each other and that conformity and markers of group membership are important ingredients in the evolution of human social behavior.
